# Cattle Body Detection Based on YOLOv5-EMA for Precision Livestock Farming

**DOI:** 10.3390/ani13223535

**Published:** 2023-11-15

**Authors:** Wangli Hao, Chao Ren, Meng Han, Li Zhang, Fuzhong Li, Zhenyu Liu

**Affiliations:** School of Software, Shanxi Agricultural University, Jinzhong 030801, China; haowangli@sxau.edu.cn (W.H.); sxaurc@stu.sxau.edu.cn (C.R.); hanm@hdu.edu.cn (M.H.); z20213621@stu.sxau.edu.cn (L.Z.); lzysyb@sxau.edu.cn (Z.L.)

**Keywords:** cattle body detection, efficient multi-scale attention, key body parts, YOLOv5-EMA

## Abstract

**Simple Summary:**

Through cattle body detection technology, breeders can promptly identify health abnormalities in cattle. Key body parts of the cattle reflect diseases to varying degrees. For example, lameness can be determined by observing the legs, and certain viral infections can be identified through an observation of the head. The early detection of these issues allows for timely intervention measures and improves treatment efficiency. It is evident that the precise detection of cattle body parts is essential. In this study, we use a computer-vision-based deep learning technique to detect individual cattle and key body parts, including the legs and head. Our proposed method enhances the accuracy of cattle body detection.

**Abstract:**

Accurate cattle body detection is crucial for precision livestock farming. However, traditional cattle body detection methods rely on manual observation, which is both time-consuming and labor-intensive. Moreover, computer-vision-based methods suffer prolonged training times and training difficulties. To address these issues, this paper proposes a novel YOLOv5-EMA model for accurate cattle body detection. By incorporating the Efficient Multi-Scale Attention (EMA) module into the backbone of YOLO series detection models, the performance of detecting smaller targets, such as heads and legs, has been significantly improved. The Efficient Multi-Scale Attention (EMA) module utilizes the large receptive fields of parallel sub-networks to gather multi-scale spatial information and establishes mutual dependencies between different spatial positions, enabling cross-spatial learning. This enhancement empowers the model to gather and integrate more comprehensive feature information, thereby improving the effectiveness of cattle body detection. The experimental results confirm the good performance of the YOLOv5-EMA model, showcasing promising results across all quantitative evaluation metrics, qualitative detection findings, and visualized Grad-CAM heatmaps. To be specific, the YOLOv5-EMA model achieves an average precision (mAP@0.5) of 95.1% in cattle body detection, 94.8% in individual cattle detection, 94.8% in leg detection, and 95.5% in head detection. Moreover, this model facilitates the efficient and precise detection of individual cattle and essential body parts in complex scenarios, especially when dealing with small targets and occlusions, significantly advancing the field of precision livestock farming.

## 1. Introduction

In the modernized process of cattle farming, monitoring the health of cattle is of paramount importance. The goal of health monitoring is to determine cattle health by observing posture, body condition, rumen filling, eyes, hair, and other factors. Therefore, achieving more accurate and efficient cattle body detection is the first step in health monitoring. Traditional methods of cattle body detection often rely on human observation, and caretakers may assess the health status of cows through physical contact. The frequent monitoring of animals’ physical condition in a quantitative manner helps in the early identification of health abnormalities [1]. Moreover, this approach requires substantial time and effort.

With the continuous advancement of technology, there has been a gradual shift towards the utilization of physical devices for cattle identification and detection. The earliest implementations involved radio-frequency identification (RFID) devices like ear tags [2]. However, in practical applications, the limitations of RFID devices have become apparent, encompassing issues such as detachment, loss, malfunctions, and label duplication, which consequently lead to a decrease in identification accuracy [3]. Conversely, Debeshi et al. [4] tackled these challenges by employing an Internet of Things (IoT) device equipped with multiple sensors, suspended from the cattle’s neck. They employed a random forest classifier to analyze the data collected from the cattle, achieving both localization and action classification, ultimately resulting in a classification accuracy of 97%. While this approach effectively discerns cattle behaviors, it may not capture visual footage of cattle movements and lacks the verification of visual images. In some cases, the behavior classification may not accurately match the actual behavior, leading to false positives.

Furthermore, as computer vision technology continues to progress, the focus of animal detection research has gradually shifted towards visual features. Object detection, a fundamental task in computer vision, involves precisely locating and identifying objects within an image. In this context, the visual features specific to cattle serve as the foundational elements for conducting cattle object detection. This evolution reflects the growing emphasis on leveraging visual cues to enhance the accuracy and effectiveness of animal detection methodologies. Zhao et al. [5] developed a method based on background subtraction for detecting moving cow targets, enabling the detection of cows in video images. Bercovich et al. [6] proposed a computer vision tool that utilizes the tail region and contour of cows for automatic segmentation and extraction, enabling the automatic detection of cows and estimation of their body condition. Liu et al. [7] introduced a dynamic background modeling method based on a mixture of Gaussian models, along with two classification algorithms that leverage chromatic distortion and luminance distortion. This approach successfully achieves the real-time extraction of cow targets under complex background conditions. Gao et al. [8] employed a multi-feature fusion approach to capture edge features, grayscale values, and spatial positional relationships of cows. They further utilized a classifier trained with the Gentle Adaboost algorithm to achieve cow body detection.

To further enhance the performance, many researchers have proposed cattle body detection methods based on deep learning.

Tassinari et al. [9] employed the YOLOv3 [10] model, a single-stage object detector, and installed a stationary camera to capture and annotate data on indoor Holstein cows for training. Their study revealed an average detection accuracy of 64%∼66%.

Lodkaew et al. [11] collected cow datasets by installing fixed cameras in cattle farms, labeled them and then trained the cow datasets using the YOLOv4 [12] model, which ultimately achieved more than 90% mAP for detection.

Andrew et al. [13] employed the Faster R-CNN [14] model to annotate and train a dataset of aerial images of Holstein cows, achieving an impressive mAP (mean average precision) of 99.6%. While the detection mAP is high, it is relatively easier to detect this breed of cows due to the significant contrast between their body color and the ground background. However, it is worth noting that the Faster R-CNN model belongs to the two-stage object detection category, which typically requires longer training time.

Xu et al. [15] employed a quadcopter to capture images of Holstein cows in different areas, including open pastures and fenced feeding grounds. They utilized the Mask R-CNN [16] model to label and train the captured dataset. Ultimately, they achieved a 96% mAP for cattle body object detection in open pastures and a 94% mAP for cattle body object detection in feeding grounds. Moreover, Xiao et al. [17] employed an improved Mask R-CNN model to train the cow dataset gathered from the barn, resulting in a remarkable achievement of 97.39% mAP. However, it is important to note that this method shares the same concern as Faster R-CNN, as both belong to the category of two-stage object detection, resulting in relatively slower training speeds.

Li et al. [18] realized the automatic recognition of yaks in field images by using random erasure and region-visibility prediction (RERP) methods based on part-based convolutional networks (PCN). However, since PCN uses a random erasure mechanism to model partial occlusion, it results in it being ineffective for detecting targets with more occlusion.

In practical livestock farming, it has been observed that key anatomical regions of cattle can be utilized for disease monitoring. For instance, issues in the leg region, such as hoof problems, may lead to reduced milk production in cows [19] and impact reproductive performance [20]. Similarly, at the head position, it is possible to observe the rumination behavior of cattle [21]. Moreover, head-related information can also be employed for individual identification [22,23]. Furthermore, the relative positioning of key anatomical regions on cattle can reflect their behavioral patterns [24]. Therefore, the detection of these key anatomical regions on cattle proves to be highly essential within the context of livestock farming.

In their study, Wu et al. [25] conducted research on lameness detection in dairy cows. They began by utilizing the YOLOv3 model to identify the leg positions of cows in different video frames. This approach successfully mitigated diverse disturbances, including occlusion, lighting variations, and proximity to objects, resulting in a notable leg position detection mAP of 93.73%. The relatively positive detection outcomes can be attributed to the fact that their experimental dataset exclusively centered on scenarios involving the detection of a single cow.

Efficient Multi-Scale Attention(EMA) [26] is an attention mechanism that utilizes a universal convolutional approach to prevent dimension reduction, effectively overcoming the constraints of conventional channel-wise dimension reduction modeling. This mechanism stands out for its flexibility and lightweight nature, allowing not only the reduction of computational expenses but also the effortless integration into diverse computer vision tasks.

By leveraging the merits of the aforementioned methods, this paper proposes the integration of an EMA attention module into the YOLOv5 network model. The aim is to achieve and enhance the detection performance of individual cattle and specific key body parts. To validate the effectiveness of the proposed model, several comparative experiments were designed to compare the performance differences between different models and YOLOv5-EMA. In summary, the key innovations of this paper encompass the following:

- From the outset, we curated an demanding dataset centered around cattle, containing a total of 8024 images. These images were captured across multiple successive days using diverse mobile devices, covering a range of angles and featuring a diverse group of 113 cattle with varying genders and ages. As a consequence of the variations in capture time and angles, these images also encompass dynamic changes in lighting, overlaps, occlusions, and other factors.
- We propose a novel cattle detection model called YOLOv5-EMA. This model improves upon YOLOv5 by incorporating the EMA attention mechanism module into the C3 module of the Backbone. The EMA attention mechanism module integrates channel attention and spatial attention units, enabling YOLOv5-EMA to focus on key regions in the image for detection and extract more comprehensive, powerful, and discriminative features. In terms of cattle detection, the YOLOv5-EMA model achieves an overall improvement of 1.0% in average precision (mAP@0.5) compared to existing models, with a 0.5% improvement in individual detection average precision (mAP@0.5). For the detection of key body parts, the average precision (mAP@0.5) for legs improves by 1.5%, and for the head, it improves by 1.1%.
- To validate the performance of the YOLOv5-EMA model, we conducted several experiments. These experiments encompassed overall comparisons of different models, comparisons of different models on individual and specific key body parts, comparisons of different attention modules, and comparisons of the EMA attention module at different positions. The aim of these experiments was to demonstrate the good performance of the YOLOv5-EMA model.

## 2. Materials and Methods

### 2.1. Dataset

**Figure 1:**
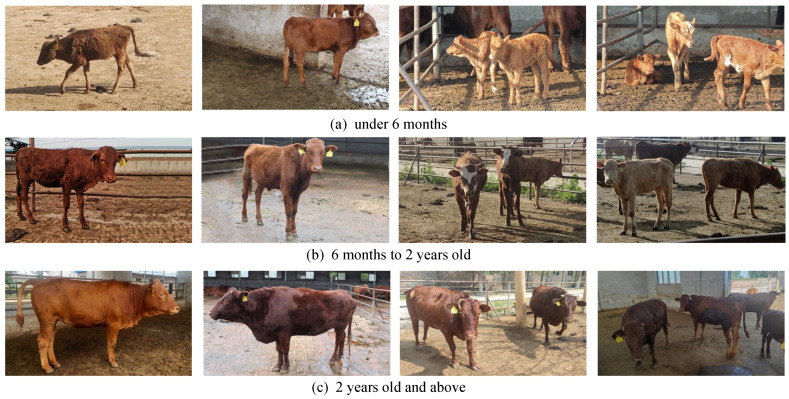
The collected data samples for cattle body detection.

The dataset was collected from the Jinnan Cattle Genetic Resource Gene Protection Center in Yuncheng City. The data collection period spanned from July to October 2021, and the subjects included healthy Jinnan cattle. Canon EOS 1300D cameras (Canon, Tokyo, Japan) and SEA-AL10 mobile phones (Huawei, Guangdong, China) were utilized to capture images from various angles between 7:00 and 20:00, encompassing different weather conditions, such as sunny, cloudy, and rainy days. The cattle were categorized into three age groups: calves from birth to 6 months old, young cattle from 6 months to 2 years old, and adult cattle over 2 years old. Some image datasets are shown in the Figure 1. All photographs were taken in natural environments, resulting in a dataset with diverse and complex scenes.

**Figure 2:**
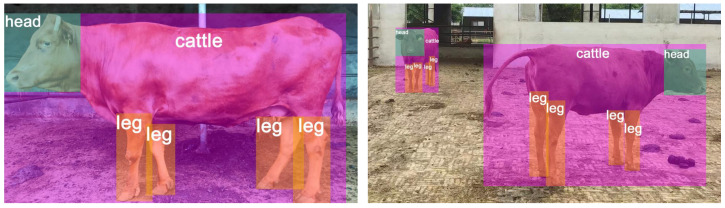
Some examples of annotated images.

To obtain an effective cattle detection model, we followed several steps for the collected images of 113 cattle. Firstly, we manually filtered out highly repetitive and blurry images from the sequentially captured images. Using the Labelimg data annotation tool, we annotated the individual, head, and leg parts separately and saved the annotated data as TXT files. These processes resulted in a dataset comprising 8024 images, with some examples of annotated images shown in Figure 2.

In the cattle body detection task, Qiao et al. [27] randomly partitioned the dataset into training and testing sets with a ratio of 7:3. This partitioning ensures that the model is trained on sufficient data, while also providing enough data to evaluate the model’s performance on unprecedented data and assess its generalization ability. The training and testing sets should exhibit similar data and feature distributions to ensure that the features learned by the model during training can be effectively applied to the testing set, thereby achieving accurate detection results. Therefore, we employ a random partitioning method for the training and testing sets. According to Table 1, To evaluate the model’s performance, we split the dataset into a training set and a test set in a 7:3 ratio, with 5617 samples for training and 2407 samples for testing.

**Figure 3:**
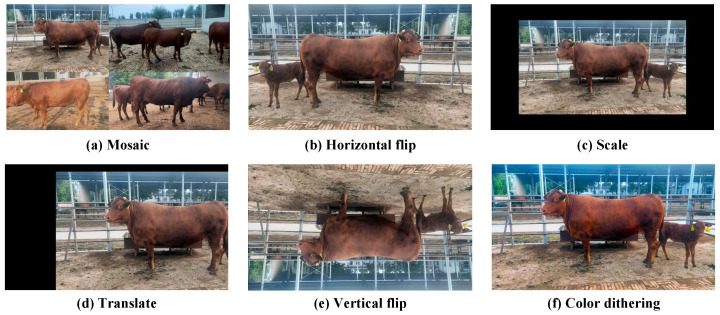
Some examples of data augmentation.

Additionally, to enhance data diversity, we employed various data augmentation techniques, including mosaic, horizontal flip, scale, translate, vertical flip, and color dithering. The color dithering of images can not only simulate different weather conditions, but also help detect and classify targets [28,29]. Some examples of data augmentation are shown in Figure 3.

### 2.2. Technical Route

**Figure 4:**
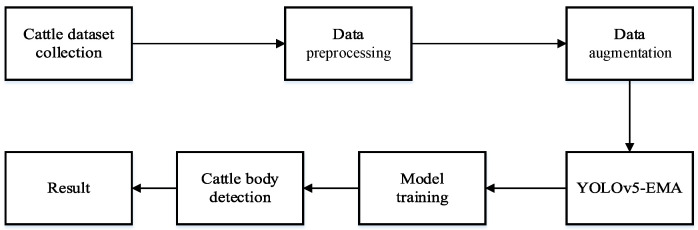
The technical route of YOLOv5-EMA for cattle body detection.

The proposed YOLOv5-EMA model adheres to the technical roadmap illustrated in Figure 4. Initially, blurry and highly repetitive images are eliminated, and the collected data undergoes preprocessing. Subsequently, diverse data augmentation techniques, including rotation, cropping, deformation, scaling, translation, and color jittering, are employed to expand the dataset and enhance its diversity, thereby improving the model’s performance. Finally, following the preprocessing stage, the images are fed into the YOLOv5-EMA model for training and detection, yielding the desired results.

### 2.3. YOLOv5-EMA

#### 2.3.1. Efficient Multi-Scale Attention (EMA)

**Figure 5:**
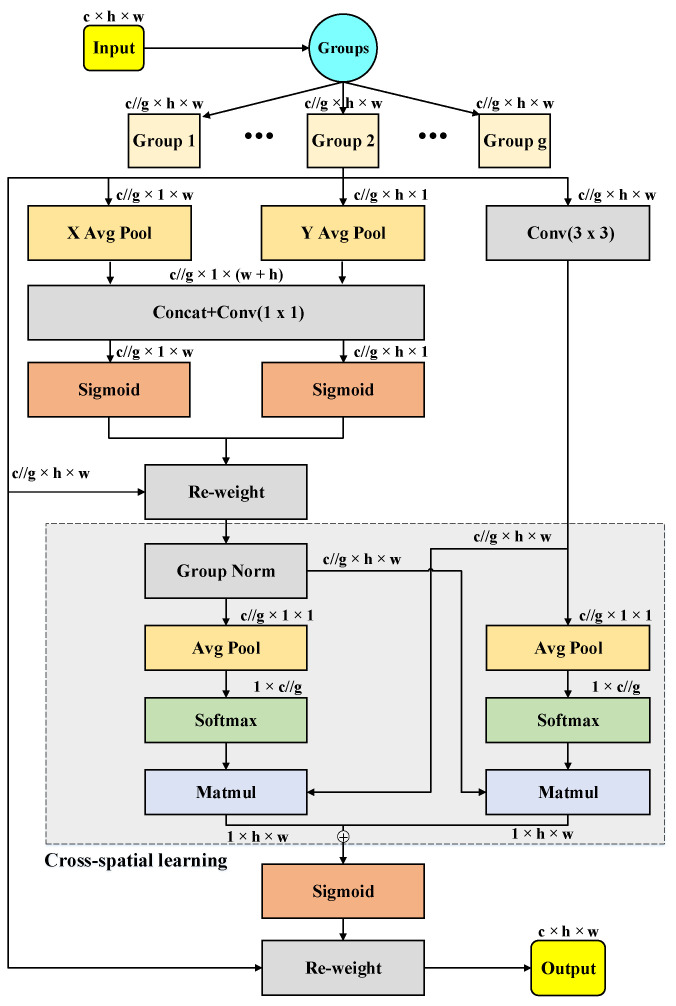
The structure of the Efficient Multi-scale Attention Module. TheDifferent colors are employed in the figure to represent distinct functional modules.

The Efficient Multi-Scale Attention (EMA) mechanism is a parallel attention mechanism used in computer vision tasks to enhance model performance and processing speed. In contrast to traditional Convolutional Neural Networks (CNNs), EMA employs a parallel structure for processing input data. This parallel convolution enables faster training of the model when dealing with large-scale data and enhances accuracy by allowing the parallel processing of features at different scales. The structure of EMA is depicted in detail in Figure 5. In Figure 5, “g” denotes the divided groups, “X Avg Pool” signifies the 1D horizontal global pooling, and “Y Avg Pool” denotes the 1D vertical global pooling, respectively.

The EMA attention mechanism first divides the input feature map $X\in R^{C\times H\times W}$ into G groups along the channel dimension, aimed at generating multiple sub-features to facilitate the capture of diverse semantic information; each group can be indicated by $X=\left[X_{1},X_{2},\ldots ,X_{G-1}\right],X_{i}\in R^{C//G\times H\times W}$. Then, EMA utilizes parallel sub-networks with large receptive fields to capture multi-scale spatial information. To extract attention weight descriptors, EMA incorporates three parallel pathways, with two in the 1×1 branch and one in the 3×3 branch. The 1×1 branch includes two 1D global average pooling operations, encoding information from two spatial directions. Then, the two encoded features along the vertical direction of the images are concatenated, and they share the same 1×1 convolution layer without reducing dimensionality in the 1×1 branch. After splitting the outputs of the 1×1 convolution into two vectors, two non-linear Sigmoid functions are applied to model the 2D Binomial distribution based on linear convolutions. To achieve diverse cross-channel interactive features between the two parallel routes in the 1×1 branch, the two channel-wise attention maps within each group are aggregated through simple multiplication. In contrast, the 3×3 branch employs a single 3×3 convolutional kernel to capture multi-scale feature representations. Consequently, EMA not only encodes inter-channel information to adjust the importance of various channels but also retains precise spatial structural details within those channels.

Furthermore, within the EMA mechanism, a cross-spatial information aggregation strategy is implemented to handle feature interactions. Specifically, 2D global average pooling is utilized for the extraction of global spatial information from the 1×1 branch outputs. In parallel, the outputs from the 3×3 branch undergo direct adjustments to align with the corresponding dimensional structure just prior to the joint activation mechanism encompassing channel features. To ensure computational efficiency, the Softmax function is employed at the outputs of 2D global average pooling. By conducting matrix dot-product operations on the results of the previously mentioned parallel processing stages, the initial spatial attention map is generated. This methodology proficiently consolidates spatial information spanning diverse scales within the same processing stage. Similarly, 2D global average pooling is harnessed to embed global spatial information within the 3×3 branch. The output of the 1×1 branch is adapted to match the corresponding dimensional configuration just before the joint activation mechanism involving channel features. As a result, the second spatial attention map is obtained, which conserves precise spatial positional information. Ultimately, the output feature map within each group undergoes further processing via a Sigmoid function.

To sum up, the EMA attention mechanism is a parallel attention mechanism employed in computer vision tasks. Its primary objective is to aid the model in capturing the interaction between features at different scales, thereby enhancing the model’s performance.

#### 2.3.2. The Detailed Architecture of YOLOv5-EMA Model

**Figure 6:**
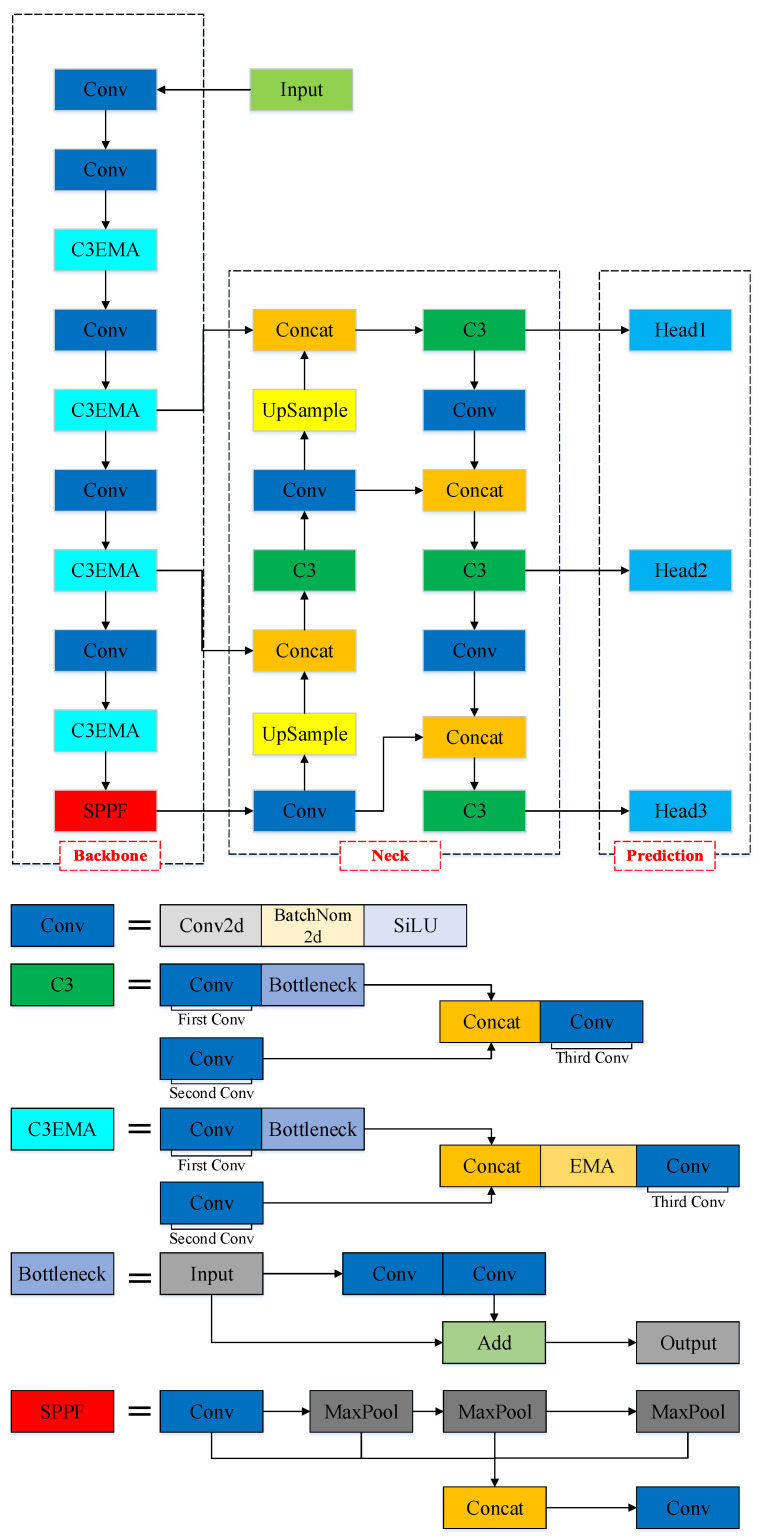
The pipeline of the proposed YOLOv5-EMA. Different colors are employed in the figure to represent distinct functional modules.

The YOLOv5-EMA model is proposed by integrating the EMA attention mechanism into the YOLOv5 backbone network. Specifically, the EMA attention module incorporates local features from multiple input sources and enhances the model’s performance through parallel processing and self-attention mechanisms. This integration significantly improves the accuracy and effectiveness of the model while preserving the excellent feature representation capability of YOLOv5. The design enhances the model’s robustness and enables the network to learn more comprehensive features. Figure 6 illustrates the architecture of the novel YOLOv5-EMA model.

Specifically, the YOLOv5-EMA network architecture comprises three main components: the backbone network, the neck structure, and the head prediction structure. In the following sections, we will provide a comprehensive description of each component.

##### Backbone

The backbone network serves as the foundation for extracting image features. Its core purpose is to transform the raw input image into a series of layered feature maps, which are then employed for object detection tasks. The backbone network consists of several crucial components, encompassing convolutional (Conv) modules, C3 modules, C3EMA modules, and the Spatial Pyramid Pooling Fusion (SPPF) module.

Conv: The Conv module is composed of a Conv2d layer, a BatchNorm2d layer, and employs the Sigmoid Linear Unit (SiLU) activation function. Within this module, the Conv2d layer carries out the convolution operation, employing a series of learnable filters to the input feature map to extract local patterns and features. Simultaneously, the BatchNorm2d layer normalizes the output from the Conv2d layer, guaranteeing steady and uniform feature representation throughout the training process. After that, the output of the BatchNorm2d layer undergoes an element-wise application of the SiLU activation function. By incorporating this activation function, non-linearity and smoothness are introduced to the feature activations. As a result, the model’s ability to grasp intricate patterns is heightened, leading to an overall enhancement in performance. The defination of SiLU is presented in the following:

(1) SiLU(x)=x×sigmoid(x)

Collectively, these units within the Conv module collaborate synergistically to extract and process features within the YOLOv5-EMA model.

C3: The C3 module plays a pivotal role within the YOLOv5-EMA network, primarily focused on extending network depth and broadening the receptive field to amplify feature extraction capabilities. The C3 module achieves this by establishing cross connections between different layers of the network.

The C3 module is composed of three Conv blocks. The first Conv block employs a stride of 2, resulting in a reduction of feature map dimensions by half. The intent behind this design is twofold: to expand the network’s receptive field and to alleviate computational demands. By downsizing the feature map dimensions, the network can focus more intently on the comprehensive object information, thereby bolstering the effectiveness of feature extraction. The subsequent two Conv blocks utilize a stride of 1. The objective of this design is to maintain the spatial resolution of the feature map, thereby enhancing the preservation of localized object information. Concurrently, the central role of these two convolutional blocks is to additionally extract features, consequently augmenting the network’s depth and receptive field. Moreover, the Bottleneck comprises two Convolutional layers. Each Conv block within the C3 module employs 3×3 convolutional kernels. Between these Convolutional blocks, Batch Normalization (BN) layers and LeakyReLU activation functions are seamlessly integrated, fostering heightened model stability and improved generalization performance.

C3EMA: The C3EMA module, an extension of the C3 framework, incorporates an EMA unit just prior to the third Convolutional block in C3. This integration empowers the C3EMA module to create interconnected relationships between channels and spatial positions, all while maintaining the spatial resolution of the feature maps. Consequently, it facilitates the implementation of cross-spatial learning. Additionally, the module takes advantage of the extensive receptive field provided by parallel sub-networks to efficiently gather multiscale spatial information, effectively enhancing its feature extraction capability.

SPPF: The SPPF module is a common pooling component found in convolutional neural networks, aimed at endowing the neural network with adaptability to spatial and positional variations in input data, thereby enhancing its recognition performance. Its fundamental idea revolves around applying multiple different scales of receptive fields to the same image, thus capturing multi-scale feature information. Within the SPPF module, the initial step involves subjecting the input feature map to various scales of pooling operations, resulting in an array of feature maps with distinct sizes. Subsequently, these feature maps are concatenated together and subjected to dimensionality reduction through fully connected layers, ultimately yielding a fixed-dimensional feature vector.

##### Neck

Within YOLOv5-EMA architecture, the “neck” component is used for fusing features of different scales. This module in YOLOv5-EMA is referred to as an FPN (Feature Pyramid Network), with the primary goal of integrating shallow-level features from the backbone network with deeper semantic features to generate a more comprehensive feature representation. By combining shallow-level features with deep semantic features, the FPN captures features at various levels, thereby providing multiple scales and rich contextual information, which helps enhance the accuracy and robustness of object detection.

##### Head

The head is a component employed to perform object detection on the feature pyramid. It comprises a series of convolutional layers, pooling layers, and fully connected layers, among other elements. In the YOLOv5-EMA model, the primary role of the detection head module is to conduct multi-scale object detection on the feature maps extracted from the backbone network.

#### 2.3.3. The Loss Function

Designing an efficient loss function is paramount for refining a cattle body detection model. Typically, the loss function comprises three fundamental components: locality loss ($L_{loc}$), category loss ($L_{cls}$), and confidence loss ($L_{conf}$). Specifically, the primary objective of the locality loss ($L_{loc}$) is to enhance the precision of cattle body instance localization. This is achieved by penalizing any discrepancies between the predicted and actual bounding box coordinates, thereby compelling the model to accurately detect cattle body parts within the image.

Concurrently, the category loss ($L_{cls}$) plays a crucial role in ensuring the accurate classification of cattle body instances. This is achieved by imposing penalties for disparities between the predicted class labels and the actual ground truth labels. As a result, the model’s capacity to correctly and effectively categorize cattle bodies within the scene is enhanced, leading to notable improvements in overall classification accuracy.

The confidence loss ($L_{conf}$) places significant emphasis on the assigned confidence scores for each detection. Its primary objective is to penalize inaccurate confidence predictions, effectively allowing the model to allocate higher scores for reliable detections and lower scores for false positives.

By incorporating these three loss function terms, the training process is guided effectively, leading to the refinement of the cattle body detection and ultimately improving its localization accuracy. The loss function employed in YOLOv5-EMA is defined as follows:

(2) $$Loss=L_{loc}+L_{cls}+L_{conf}$$

##### The Locality Loss $L_{loc}$

This loss function serves as an evaluation metric for assessing the network’s precision in predicting the positions of target bounding boxes. YOLOv5-EMA utilizes the CIOU loss [30] for computing the regression loss of bounding boxes. Its primary objective is to aid the network in acquiring the capability to accurately predict the bounding boxes of target objects. The specific formula is provided below:

(3) $$L_{loc}=L_{CIoU}=1-IoU+\frac{\rho^{2}\left(b,b^{gt}\right)}{c^{2}}+\alpha v$$

where IoU represents the Intersection over Union and defined:

(4) $$IoU=\frac{\left|A\cap B\right|}{\left|A\cup B\right|}$$

The IoU (Intersection over Union) metric assesses the extent of overlap between the predicted bounding box (referred to as *A*) and the ground truth bounding box (referred to as *B*). It quantifies how closely the predicted region aligns with the ground truth region. Specifically, IoU values span from 0 to 1, where a score of 1 indicates a perfect alignment between the predicted and ground truth regions, while a score of 0 indicates complete the absence of overlap.

The center points of the predicted and ground truth bounding boxes are denoted as *b* and $b^{gt}$, respectively. The Euclidean distance between these two center points is represented by ρ. The diagonal length of the minimum enclosing rectangle that can encompass both the predicted and ground truth bounding boxes is denoted as *c* to quantify the consistency of aspect ratios. Additionally, there is a weight function denoted by α. The definitions of both *v* and α are provided in the following sections.

(5) $$v=\frac{4}{\pi^{2}}\left(arctan\frac{w^{gt}}{h^{gt}}-arctan\frac{w}{h}\right)$$

(6) $$\alpha =\frac{v}{1-IoU}+v$$

Here, $w^{gt}$ and $h^{gt}$ correspond to the width and height of the ground truth bounding box, while *w* and *h* refer to the width and height of the predicted bounding box.

##### The Category Loss $L_{cls}$

This category loss is defined as follows:

(7) $$L_{cls}=-\frac{1}{N}\sum [t\cdot log(p)+\left(1-t\right)\cdot log(1-p)]$$

where *N* represents the number of object categories, *p*/*t* signifies the predicted/real category label for a certain object within the bounding boxes.

##### The Confidence Loss $L_{conf}$

The confidence loss is determined using the following equations:

(8) $$\begin{array}{c} L_{conf}=-\sum_{i=0}^{s^{2}}\sum_{j=0}^{B}I_{i,j}^{obj}\left[{\bar{C}}_{i}^{j}log\left(C_{i}^{j}\right)+\left(1-{\bar{C}}_{i}^{j}\right)log\left(1-C_{i}^{j}\right)\right]\\ -\lambda_{noobj}\sum_{i=0}^{s^{2}}\sum_{j=0}^{B}I_{i,j}^{noobj}\left[{\bar{C}}_{i}^{j}log\left(C_{i}^{j}\right)+\left(1-{\bar{C}}_{i}^{j}\right)log\left(1-C_{i}^{j}\right)\right] \end{array}$$

$s^{2}$ represents a grid of size s×s, and *B* signifies the number of bounding boxes predicted for each grid. The indicator $I_{i,j}^{obj}$ takes on the value 1 when a target is detected in the *j*th box, and 0 otherwise. Conversely, the indicator $I_{i,j}^{noobj}$ is 0 when a target is present in the *j*th box, and 1 otherwise. $\bar{C}$ and *C* represent the confidence values of the predicted and annotated boxes, respectively. Additionally, $\lambda_{noobj}$ serves as a hyperparameter used to adjust the relative importance of these two terms.

#### 2.3.4. Evaluation Metric

To effectively validate the effectiveness of the proposed model, this paper leverages several evaluation metrics, including precision (Equation (9)), recall (Equation (10)), *F*1_score (Equation (11)), average precision (Equation (12)), and mean Average Precision (mAP) (Equation (13)), separately. Moreover, the utilization of Grad-CAM heatmaps offers a more intuitive insight into the decision-making process underlying the network’s prediction results. These evaluation metrics are defined as follows.

Precision denotes the proportion of true positive samples among all samples predicted as positive and can be defined as follows:

(9) P=TP/(TP+FP)

Recall represents the proportion of true positive samples among all actual positive samples and can be formulated as follows:

(10) R=TP/(TP+FN)

The *F*1_score denotes the harmonic mean of precision and recall, and its value falls between 0 and 1. This evaluation criteria provides a balanced measure of a model’s performance, taking into account both precision and recall, making it a useful metric for evaluating classification models.

(11) $$F1\_score=\frac{2PR}{P+R}$$

Average precision (*AP*) represents the mean of precision values calculated at different recall levels. This metric offers a holistic assessment of the model’s performance across a range of recall thresholds.

(12) $$AP=\int_{0}^{1}P(R)dR$$

Finally, the mean Average Precision (mAP), serves as the average of the individual AP values computed for various classes or categories. This metric delivers a comprehensive evaluation of the model’s proficiency in object detection tasks. These evaluation metrics, collectively, gauge the model’s effectiveness and precision in the detection and quantification of objects. Typically, the results are assessed using mAP@0.5, where the value following the @ symbol signifies the threshold utilized to distinguish between positive and negative samples based on Intersection over Union (IoU).

(13) $$mAP=\frac{\sum_{1}^{n}\left(AP\right)}{n}$$

Grad-CAM [31] is a visualization technique utilized to interpret the predictions made by deep neural networks in tasks such as image classification and object detection. Grad-CAM, short for Gradient-weighted Class Activation Mapping, calculates the gradient weights of a particular class with respect to each position or feature map within the network. This process generates a heatmap that illustrates the significance of each position or feature map in relation to the prediction outcome.

#### 2.3.5. Experimental Setup

This study was conducted on a Linux Ubuntu 18.04 operating system, utilizing the PyTorch deep learning framework. The hardware utilized for the experiments included an Intel Core i7 7800X CPU, NVIDIA GeForce GTX TITAN XP GPU, and 128 GB of memory. Concerning the model training, the experiment consisted of 100 training epochs, with a batch size of 16. The initial learning rate was set to 0.01, and a learning rate momentum of 0.937 was applied. The input image size is 640 × 640. Additional details about the hardware and software configuration employed in the experiments are presented in Table 2.

The training process of the YOLOv5-EMA is presented in Algorithm 1.
**Algorithm 1:** YOLOv5-EMA model training
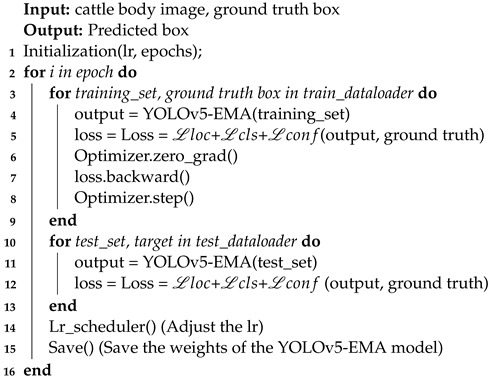


## 3. Experimental Results and Analyses

In this section, we will provide a comprehensive presentation of the experimental results and engage in corresponding discussions. The experiments are organized into several parts, encompassing a holistic comparison of different models, individual and specific key body part comparisons across various models, assessments of distinct attention modules, and an evaluation of the EMA attention module’s performance at different positions. In summary, the objective of these evaluations is to ascertain the superiority of YOLOv5-EMA.

Throughout this study, the term “cattle” refers to individual cattle, “head” denotes the head part, and “leg” indicates the leg part.

### 3.1. Comparison of Different Models

In order to validate the effectiveness of the YOLOv5-EMA model, we conducted a comparative analysis with several other models, assessing their performance in both overall cattle and specific key body parts detection. These models include Faster R-CNN, SSD [32], YOLOv2 [33], YOLOv3, YOLOv4, and YOLOv5. The comprehensive results of this comparative analysis are summarized in Table 3.

Table 3 demonstrates that the YOLOv5-EMA model exhibits good performance across a majority of evaluation metrics. Specifically, using YOLOv5-EMA, we achieved a precision of 94.8%, a recall of 90.3%, an *F*1 score of 92.5%, and an mAP@0.5 of 95.1%. These results undeniably confirm the effectiveness of the YOLOv5-EMA model.

Although the YOLOv5-EMA model slightly lags behind Faster R-CNN in terms of Recall, it outperforms Faster R-CNN in Precision, *F*1 score, and mAP@0.5. Notably, Faster R-CNN operates as a two-stage detection model, which entails a comparatively slower training speed and detection efficiency. Consequently, the YOLOv5-EMA model emerges as a frontrunner in overall detection performance.

**Figure 7:**
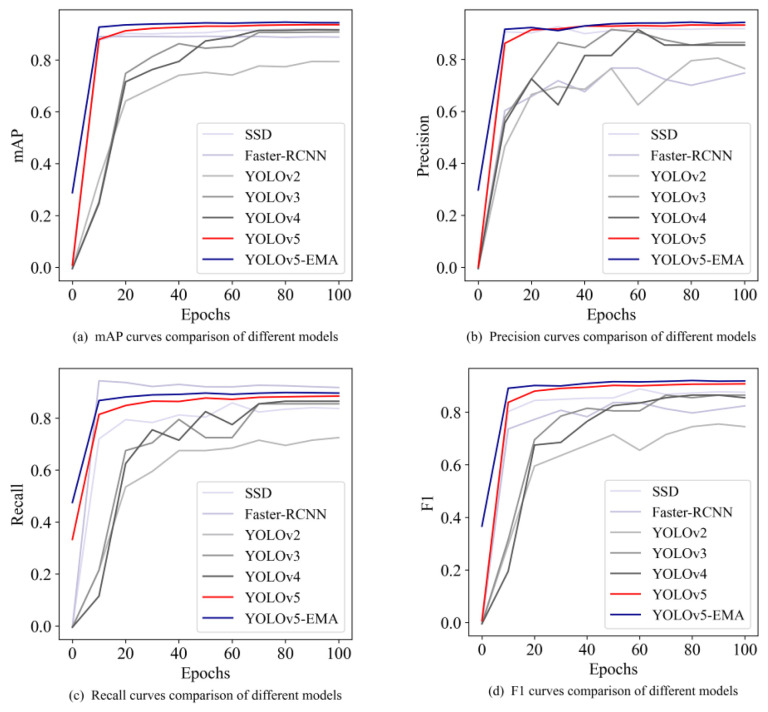
The comparison curves of different models, including SSD, Faster R-CNN, YOLOv2, YOLOv3, YOLOv4, YOLOv5, and YOLOv5-EMA, are depicted in the four subplots. (**a**) The curves of mAP under different iterations. (**b**) The curves of Precision under different iterations. (**c**) The curves of Recall under different iterations. (**d**) The curves of *F*1-score under different iterations.

Figure 7 illustrates the comparison results of different models across various iterations. The curves representing mAP, Precision, and *F*1-score of YOLOv5-EMA consistently outperform those of Faster-RCNN, SSD, YOLOv2, YOLOv3, YOLOv4, and YOLOv5. This indicates that YOLOv5-EMA exhibits good performance in terms of mAP, Precision, and *F*1-score compared to the other models.

### 3.2. Comparison of Different Models on Individual Cattle and Specific Key Body Parts

To assess the effectiveness of the YOLOv5-EMA model across different critical aspects of cattle anatomy, we will analyze its performance on individual cattle, as well as on their legs and heads. The results of these performance analyses for various models can be found in Table 4.

Table 4 illustrates that the YOLOv5-EMA model exhibits good performance in the target detection of individuals and key body parts in most evaluation indicators. Specifically, YOLOv5-EMA achieved an mAP@0.5 of 94.8% in individual cattle detection, an mAP@0.5 of 94.9% in leg object detection, and an mAP@0.5 of 95.5% in head object detection. Table 4 indicates that the YOLOv5-EMA model outperforms all other models on the mAP@0.5 metric across various body parts. This result validates the effectiveness of the YOLOv5-EMA model in detecting key cattle body parts.

**Figure 8:**
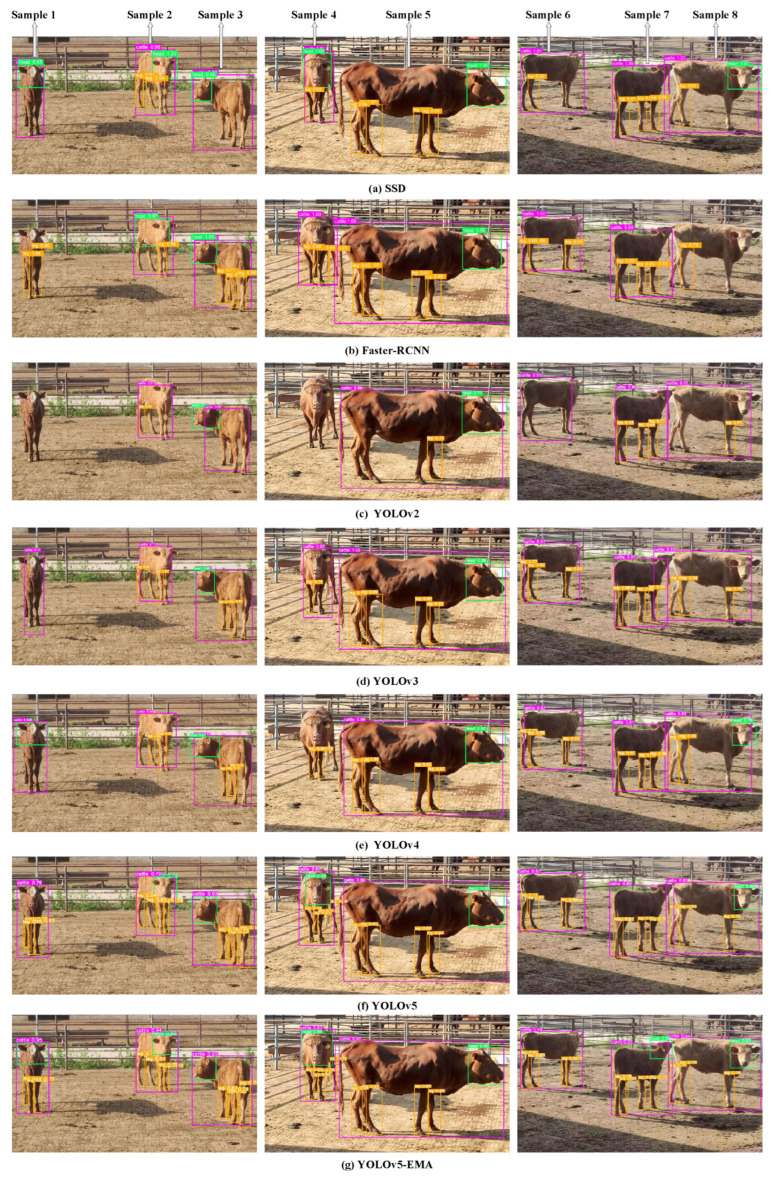
The qualitative comparison of different models, including SSD, Faster R-CNN, YOLOv2, YOLOv3, YOLOv4, YOLOv5, and YOLOv5-EMA, respectively. The purple, orange, and green rectangular boxes in the figure represent the detection boxes for individual cattle, legs, and heads, respectively.

Additionally, the qualitative comparison results of various models, such as Faster R-CNN, SSD, YOLOv2, YOLOv3, YOLOv4, YOLOv5, and YOLOv5-EMA, are illustrated in Figure 8. For clarity, we have labeled the eight cattle from left to right in the Figure 8 as Sample 1 to Sample 8. The detailed description of the visualization of the detection results for each sample is as follows.

Sample1: For Sample1, in terms of individual cattle detection, Faster-RCNN and YOLOv2 failed to detect the target. Although SSD, YOLOv4, YOLOv3, and YOLOv5 detected the target, there were errors in the detection bounding boxes. The best detection performance was achieved by YOLOv5-EMA. Regarding head detection, Faster-RCNN, YOLOv2, YOLOv3, and YOLOv5 did not detect the target. While SSD detected the target, the right ear part was missed within the detection bounding box. YOLOv4 and the YOLOv5-EMA model exhibited the best detection results. For leg detection, due to the cow’s standing angle, one leg was completely obscured, and only three legs were visible in reality. SSD, YOLOv2, YOLOv3, and YOLOv4 failed to detect the target. Faster-RCNN detected two legs, and YOLOv5 detected four legs (with false positives). However, both of these models had errors in their detection bounding boxes. The best leg detection performance was achieved by YOLOv5-EMA.

Sample2: Concerning Sample2, in terms of individual cattle detection, SSD, Faster-RCNN, YOLOv2, YOLOv3, YOLOv4, and YOLOv5 all detected the target; however, there were errors in the detection bounding boxes, particularly with the right side of the body extending beyond the bounding box. The most outstanding detection performance was achieved by YOLOv5-EMA. In head detection, YOLOv2, YOLOv3, and YOLOv4 failed to detect the target. Although Faster-RCNN and YOLOv5 detected the target, the former incorrectly identified a portion of the body as the head, and the latter had the left ear detected outside the bounding box. SSD and the YOLOv5-EMA model exhibited the best head detection performance. For leg detection, Faster-RCNN and YOLOv2 only detected one leg, while SSD, YOLOv3, and YOLOv4 detected two legs. However, all of these models had errors in their detection bounding boxes relative to the true target. YOLOv5 and YOLOv5-EMA demonstrated the most promising leg detection performance.

Sample3: Regarding Sample3, concerning the individual cattle detection, SSD, Faster-RCNN, YOLOv2, YOLOv3, YOLOv4, YOLOv5, and YOLOv5-EMA were all able to detect the target. However, there were slight inaccuracies in their detection bounding boxes. Notably, YOLOv5-EMA exhibited the smallest errors. In head detection, although YOLOv2 and YOLOv4 also detected the target, they mistakenly included environmental elements within the detection boxes. Conversely, the SSD, Faster-RCNN, YOLOv3, YOLOv5, and YOLOv5-EMA models performed well in head detection. As for leg detection, SSD and YOLOv2 failed to detect the target, YOLOv3 detected two legs, YOLOv4 detected three legs, and Faster-RCNN, YOLOv5, and YOLOv5-EMA all detected four legs. Among these, YOLOv5 and YOLOv5-EMA achieved the best leg detection performance, while other models had some errors in their detection bounding boxes compared to the ground truth target.

Sample4: For sample4, regarding the individual cattle detection, YOLOv2 and YOLOv4 failed to detect the target. Although other models successfully detected the target, there were some inaccuracies in their detection bounding boxes. Only YOLOv5-EMA achieved the smallest error in this regard. Regarding head detection, Faster-RCNN, YOLOv2, YOLOv3, and YOLOv4 failed to detect the target. While SSD and YOLOv5 detected the target, their detection boxes did not fully encompass the left cattle horn. Only the YOLOv5-EMA model demonstrated the best head detection performance. As for leg detection, YOLOv2 failed to detect the target, SSD and YOLOv3 detected one leg, YOLOv4 detected two legs, and Faster-RCNN detected three legs. Although YOLOv5 and YOLOv5-EMA successfully detected all four legs, YOLOv5-EMA performed better in detecting the right hind leg.

Sample5: For sample5, in terms of individual cattle detection, SSD failed to detect the target. Although other models successfully identified the target, their detection boxes exhibited some inaccuracies, particularly around the tail and mouth of the cattle. Only YOLOv5-EMA managed to precisely encompass the entire cattle within the detection box. Regarding head detection, all models successfully recognized the target, but Faster-RCNN, YOLOv2, YOLOv3, and YOLOv4 showed some disparities in their detection boxes compared to the actual objects. In terms of leg detection, YOLOv2 detected only one leg, while the other models detected all four legs. However, the targets were not entirely enclosed within the detection boxes for the latter models. Conversely, YOLOv5-EMA accurately enclosed the entire target within the detection box.

Sample6: For cattle labeled as sample6, regarding individual detection, all models succeeded in detecting the cattle; however, disparities in their detection boxes arose, especially around the right hind leg and the cattle’s head. Precisely encompassing the entire target within their detection frames was achieved exclusively by SSD and YOLOv5-EMA. Concerning head detection, given the cattle’s standing angle, none of the models successfully detected the target. Regarding leg detection, YOLOv2 failed to detect any legs, SSD identified one leg, whereas Faster-RCNN, YOLOv3, and YOLOv4 detected three legs. The YOLOv5 and YOLOv5-EMA models were the only ones to accurately enclose the entire target within their detection frames.

Sample7: In the case of cattle labeled as sample7, all models successfully detected the individual target. Nevertheless, there were disparities in their detection boxes compared to the actual target, particularly in the vicinity of the cattle’s mouth. Notably, only the YOLOv5-EMA model demonstrated the least error in this aspect. When it came to head detection, solely the YOLOv5-EMA model achieved a successful target detection. As for leg detection, YOLOv2 identified one leg, whereas SSD, Faster-RCNN, YOLOv2, and YOLOv4 detected three legs. Although the YOLOv3, YOLOv5, and YOLOv5-EMA models recognized four legs, only the YOLOv5 and YOLOv5-EMA models accurately enclosed the entire target within their detection frames. Other models exhibited inaccuracies in their detection boxes relative to the true target.

Sample8: For sample8, Faster-RCNN encountered difficulties in successfully detecting the target, whereas other models managed to identify the target. However, these models exhibited certain inaccuracies in their detection boxes, particularly around the tail and ears of the cattle. Only the YOLOv5-EMA model succeeded in fully encompassing the target within its detection box. In the realm of head detection, Faster-RCNN, YOLOv2, and YOLOv3 struggled to detect the target effectively, whereas YOLOv4 and YOLOv5, while detecting the target, also displayed inaccuracies in their detection boxes, particularly around the ears. It was only SSD and the YOLOv5-EMA model that could accurately enclose the target within their detection boxes. Concerning leg detection, due to the angle at which the cattle were positioned, one leg remained entirely obscured, leaving only three legs visible in reality. Among these scenarios, SSD, Faster-RCNN, and YOLOv2 identified one leg, while YOLOv3 and YOLOv4 identified two legs. Although YOLOv3 detected three legs, it was plagued by inaccuracies in its detection boxes. In contrast, YOLOv5 and the YOLOv5-EMA model successfully identified all three legs with the highest level of precision.

Above all, Figure 8 illustrates that YOLOv5-EMA surpasses other models by generating more precise predicted bounding boxes, particularly in scenarios involving occlusion and other demanding situations.

In the YOLOv5-EMA model, the EMA attention module first leverages the large receptive fields of parallel sub-networks to gather multi-scale spatial information. It subsequently establishes mutual dependencies between various spatial positions, facilitating cross-space learning. This empowers the YOLOv5-EMA model to consolidate more comprehensive feature information when detecting individual cattle and specific key areas such as the head and legs. As a result, it significantly enhances the model’s feature extraction capabilities. The experimental results confirm that the YOLOv5-EMA model performs well in tasks related to individual detection and the identification of specific key areas, thus validating its good performance compared to other models.

### 3.3. Evaluating the Effectiveness of the EMA Unit

To assess the effectiveness of the EMA attention module, we compare two models with and without EMA modules, namely YOLOv3 and YOLOv3-EMA; YOLOv5 and YOLOv5-EMA. YOLOv5-EMA is formed by incorporating the EMA attention module into the YOLOv5 backbone. The comparison results are shown in Table 5 and Table 6.

Table 5 demonstrates that models incorporating the EMA attention module based on YOLOv3 and YOLOv5 exhibit good performance across all evaluation metrics. Specifically, with YOLOv3-EMA, we achieved a precision of 94.5%, a recall of 88.8%, *F*1 score of 91.8%, and an mAP@0.5 of 93.9%. Similarly, YOLOv5-EMA achieved outstanding results.

Table 6 illustrates that models incorporating the EMA attention module based on YOLOv3 and YOLOv5 demonstrate good performance improvements in various key parts across all evaluation metrics. To be more specific, YOLOv3-EMA achieved an mAP@0.5 of 94.2% in individual cattle detection, an mAP@0.5 of 93.3% in leg object detection, and an mAP@0.5 of 94.3% in head object detection. Comparatively, YOLOv5-EMA attained an mAP@0.5 of 94.8% in cattle object detection, which aligns with the performance of YOLOv5 models. YOLOv5-EMA achieved an mAP@0.5 of 94.9% in leg object detection, and an mAP@0.5 of 95.5% in head object detection. There was a significant improvement in the detection mAP for the data related to the legs and head.

**Figure 9:**
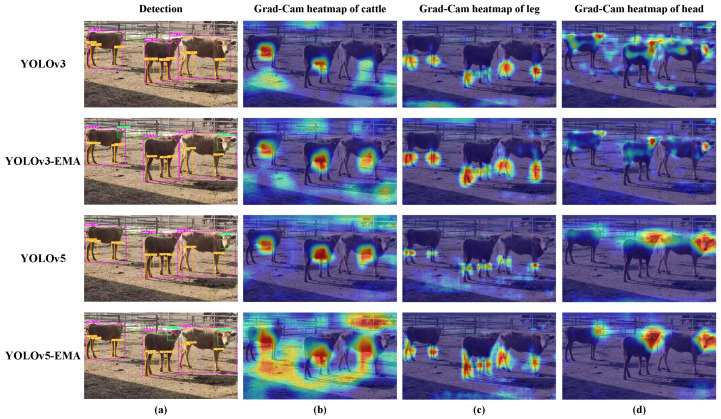
The qualitative detection results and the Grad-CAM heatmap of models with or without EMA attention unit. Subfigure (**a**) shows the qualitative detection results of different parts of the cattle body, with purple, orange, and green rectangular boxes representing the detection boxes of the cattle individual, leg, and head, respectively. Subfigures (**b**–**d**) show the Grad-CAM heatmaps of the cattle individual, leg, and head, respectively. The redder the color, the greater the contribution of the region to the model’s classification results.

Additionally, to provide readers with a more intuitive understanding, Figure 9 illustrates the qualitative comparison results and the Grad-CAM heatmap of the different models with or without EMA.

Figure 9a shows the predictive results of different models. We have observed the following phenomenon. Individual cattle detection: For individual cattle detection, YOLOv3 faced the following issues, with instances outside the detection boxes. Alternatively, for the YOLOv3-EMA model, two cattle were detected entirely within the detection boxes, and the error in the detection box for the middle cow was significantly reduced. Furthermore, for the YOLOv5 model, there were similar issues in the individual cattle detection, particularly for the middle and rightmost cows, where real targets were sometimes detected outside the detection boxes. In contrast, concerning YOLOv5-EMA model, the rightmost cow was consistently detected entirely within the detection box, and the error for the middle cow was significantly reduced. Cattle head detection: Regarding cattle head detection, YOLOv3 failed to detect any targets initially. Alternatively, for the YOLOv3-EMA model, two cattle heads were successfully detected, leading to a noticeable improvement in detection performance. Similarly, YOLOv5 detected two cattle heads, but the rightmost cattle’s ear part was detected outside the detection box. For the YOLOv5-EMA model, not only were the right two cattle heads detected, but the rightmost cattle head was completely contained within the detection box. Cattle leg detection: In the case of cattle leg detection, YOLOv3 encountered the following issues, include missing one leg on the leftmost cattle, occasionally detecting the legs of the middle and rightmost cows outside the detection boxes, respectively. On the other hand, for YOLOv3-EMA, all the legs of the leftmost cattle were successfully detected, and although one leg of the middle cow was still occasionally missed, the detected legs were accurately positioned within the detection box. In both YOLOv5 and YOLOv5-EMA, all cattle legs were successfully detected; YOLOv5-EMA exhibited more precise detection of the right hind leg of the leftmost cattle.

Further, Figure 9b–d present the Grad-CAM heatmaps of different models. Individual cattle detection: Concerning the individual cattle detection, YOLOv3 exhibits a poor performance on the rightmost cattle. On the contrary, YOLOv3-EMA brings about a notable shift in detection focus, transitioning from the surroundings to the abdominal region of the cattle for improved accuracy. In addition, while YOLOv5 primarily emphasizes the cattle’s abdomen, YOLOv5-EMA goes a step further by incorporating environmental awareness alongside its focus on the abdomen. Cattle head detection: For cattle head detection, the attention of YOLOv3 appears to be scattered across the head, abdomen, and tail regions. Yet, for YOLOv3-EMA, the detection focus sharply narrows down to the cow’s head region. YOLOv5 exhibits a diminished emphasis on the head of the leftmost cattle, whereas YOLOv5-EMA enhances this attention. Cattle leg detection: When it comes to cattle leg detection, YOLOv3 and YOLOv5 primarily focus their attention on the knee area of the cattle’s legs. However, in contrast, YOLOv3-EMA and YOLOv5-EMA shift their focus towards the entire cattle’s legs.

By incorporating the EMA attention module into the Backbone of YOLO series detection models, we have successfully improved the detection performance of smaller targets such as heads and legs. This enhancement is attributed to the design of the EMA attention module, which establishes a multiscale parallel subnetwork to capture long and short dependencies. Without reducing the number of channels, each parallel subnetwork constructs localized cross-channel interactions. Through this method, the model is able to fuse the feature maps from parallel subnetworks, thereby enhancing the diversity and quality of feature fusion. Upon comprehensive analysis, incorporating the EMA attention module into the backbone can notably enhance the detection performance of the YOLOv3 and YOLOv5 models.

### 3.4. Comparison of Different Attention Modules

In order to evaluate the effectiveness of the EMA attention module, we conducted a comparison of several common attention mechanisms, including ECA [34], SE [35], CBAM [36], and CA [37]. Among them, ECA and SE only contain channel attention, qhile CA and CBAM include channel and spatial attention. These attention mechanisms were integrated into the C3 module of the Backbone and subsequently incorporated into the main network of YOLOv5. The comparison results of this comparative analysis are presented in Table 7.

Table 7 demonstrates that the YOLOv5-EMA model, enhanced with the EMA attention components, exhibits the best performance across all evaluation metrics. Specifically, the model with the added EMA module achieved an mAP@0.5 of 95.1% in overall object detection, an mAP@0.5 of 94.9% in leg object detection, and an mAP@0.5 of 95.5% in head object detection. Only in the detection of individual cattle was the improvement not significant. These results validate that the model with EMA is better than models with other attention components.

**Figure 10:**
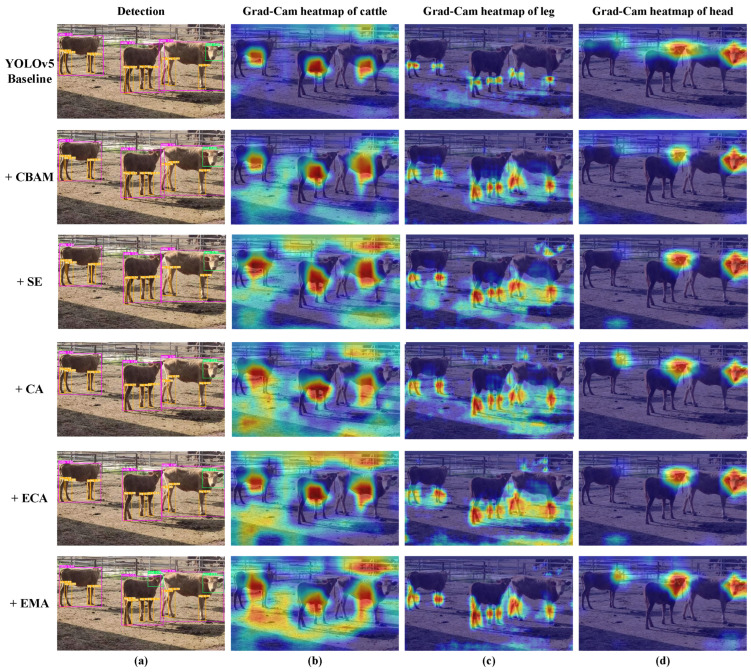
The qualitative detection results and the Grad-CAM heatmap of YOLOv5 baseline models with various attention components. Subfigure (**a**) shows the qualitative detection results of different parts of the cattle body, with purple, orange, and green rectangular boxes representing the detection boxes of the cattle individual, leg, and head, respectively. Subfigures (**b**–**d**) show the Grad-CAM heatmaps of the cattle individual, leg, and head, respectively. The redder the color, the greater the contribution of the region to the model’s classification results.

Furthermore, to offer readers a clearer and more intuitive comprehension, Figure 10 presents the qualitative detection results and the Grad-CAM heatmap of models with various attention components.

Based on Figure 10a, it can be observed that, in the individual cattle detection task, all models successfully detect three cattle. Concerning the cattle head detection, only the model with the EMA attention module successfully detects the head of the middle cattle. For the cattle leg detection, models with the CA and ECA attention components tend to miss the left hind leg of the middle cattle, whereas YOLOv5-EMA (+EMA) significantly reduces the error between the detected bounding boxes and the actual targets, resulting in a more accurate performance.

From Figure 10b–d, it can be observed that after incorporating different attention mechanisms, the generated heatmaps for individual object detection exhibit varying degrees of consideration for contextual information. In the context of leg detection, only the model with the EMA attention component focuses on the entire leg region, obtaining precise and compact heatmap regions. For head detection, models with both CA and EMA attention mechanisms successfully prioritize the head region of the leftmost cow, whereas other models lose this object to varying degrees.

Overall, the YOLOv5-EMA model exhibits good performance in detecting small targets like heads, legs, and obscured objects. EMA’s superiority can be attributed to several key factors. Unlike other attention models that concentrate solely on channel information or consider both channel and spatial information without exploring information dependencies across various spatial scales, EMA attention stands out. It effectively combines spatial and channel attention, facilitating information interaction among feature maps at different spatial scales. As a result, it significantly enhances the model’s detection performance.

### 3.5. Comparison of Models with EMA Integrated at Different Positions

**Figure 11:**
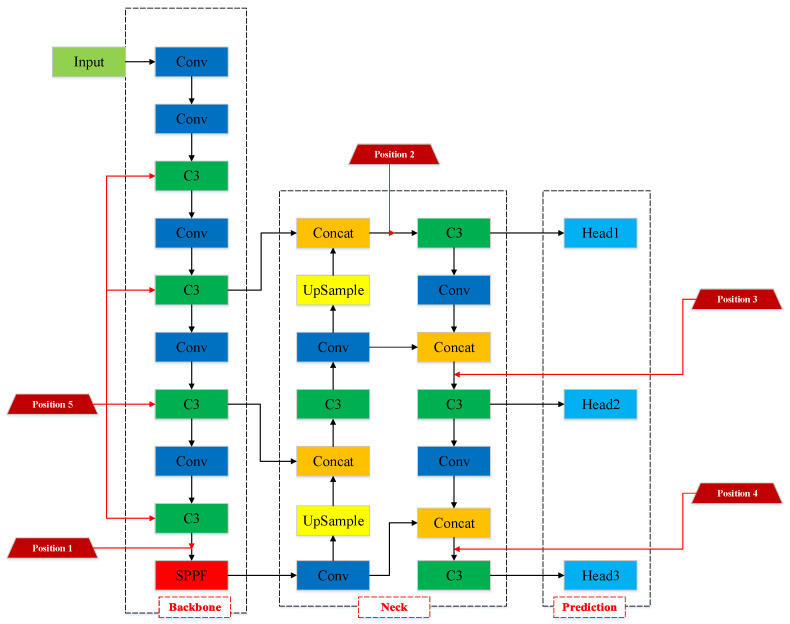
Schematic diagram of the model with the EMA attention component integrated at various positions. Different-colored rectangular boxes are used in the figure to represent different functional modules. The red trapezoidal box represents the network location where EMA attention components are integrated.

In order to determine the optimal location for integrating the EMA attention module within the Backbone, we conducted a comparative analysis using variant models where attention was positioned at five distinct locations. These positions can be described as follows: Position 1: between the last C3 module and the SPPF module in the backbone; Position2: between the C3 module in the Neck, connected to Head1 detection layer and the concat module; Position 3: between the C3 module in the Neck, connected to Head2 detection layer and the concat module; Position 4: between the C3 module in the Neck, connected to Head3 detection layer and the concat module; and Position 5: inside the C3 module within the Backbone. All of these positions are closely related to the feature extraction locations crucial for object detection. The specific placement of the EMA attention module is visually depicted in Figure 11. The experimental results involve a comparison of detection performance for individual and local key points, assessed using the mAP@0.5 metric, as presented in Table 8.

According to Table 8, it is evident that the model with the EMA attention component added at position 5 (the C3 module of the Backbone) achieves the highest mAP@0.5 values for individual, head, and leg object detection. Furthermore, it exhibits the most significant improvement in leg and head detection performance. While adding the EMA attention mechanism at other positions also substantially enhances individual object detection, it falls short in improving leg and head detection compared to the model with EMA attention at position 5. Specifically, the model that adds the EMA module at position 5 achieved an mAP@0.5 of 95.1% in overall object detection, an mAP@0.5 of 94.9% in leg object detection, and an mAP@0.5 of 95.5% in head object detection. These results confirm that Position 5 is the optimal location for integrating the EMA module.

**Figure 12:**
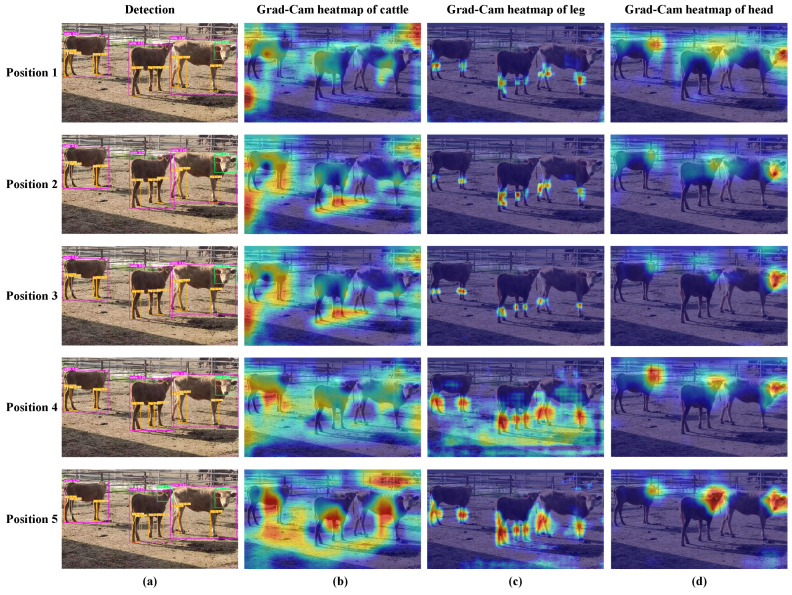
The qualitative detection results and the Grad-CAM heatmap of models with EMA attention components integrated at various positions. Subfigure (**a**) shows the qualitative detection results of different parts of the cattle body, with purple, orange, and green rectangular boxes representing the detection boxes of the cattle individual, leg, and head, respectively. Subfigures (**b**–**d**) show the Grad-CAM heatmaps of the cattle individual, leg, and head, respectively. The redder the color, the greater the contribution of the region to the model’s classification results.

Furthermore, to provide readers with a clearer and more intuitive understanding, Figure 12 presents the qualitative detection results and Grad-CAM heatmaps for models with the EMA attention component integrated at different positions.

In Figure 12a, it becomes clear that models incorporating the EMA attention mechanism from Position 1 to Position 5 can proficiently detect all three cattle during individual detection. However, when narrowing our focus to head detection, only the model with EMA integrated at Position 5 successfully identifies the head of the middle cattle. Similarly, in leg detection, only the model with EMA integrated at Position 5 accurately discerns the left hind leg of the middle cattle.

From Figure 12b–d, it is evident that, in individual cattle detection, models incorporating EMA at Positions 1, 2, 3, and 4 exhibit reduced attention towards the middle and rightmost cattle. Only the model with EMA at position 5 effectively concentrates its detection focus on the abdominal region of all three cattle. In terms of cattle leg detection, models with EMA at Positions 1, 2, and 3 primarily focus on the knee area. In contrast, models with EMA at positions 4 and 5 expand their attention area to cover the entire leg of the cattle. Concerning cattle head detection, models with EMA at positions 1, 2, 3, and 4 exhibit weaker attention towards the head of the middle cattle. Only the model with EMA at Position 5 achieves the best attention focus on the head of the middle cattle.

In summary, integrating the EMA attention mechanism at position 5 yields the most optimal detection performance. This provides compelling evidence for the effectiveness of incorporating EMA attention into the C3 module of the YOLOv5 Backbone. The primary function of the C3 module in the Backbone is to increase the network depth and receptive field, thereby enhancing feature extraction capabilities. In the experiment, we achieved improved robustness by incorporating the EMA attention mechanism within the C3 module. This allowed for cross-spatial information aggregation along different spatial dimensions, further enhancing feature representational capacity. This approach notably improved the detection performance, particularly in cases involving small targets and occlusions.

## 4. Discussion

At the beginning of our research, we investigated the application of deep learning models based on computer vision for cattle body detection [9,11,13,25]. The commonly used models included one-stage detectors, such as SSD [32], YOLOv2 [33], YOLOv3 [10], YOLOv4 [12], YOLOv5, and two-stage detectors like Faster R-CNN [14]. However, due to the two-stage detectors requiring two stages, including candidate box generation, target classification, and localization, they are slower in processing speed. Additionally, the candidate regions generated by the Region Proposal Network may not accurately capture the positions and bounding boxes of small targets. This deficiency makes them unsuitable for small-sized targets detection tasks [38]. Considering our research involved detecting small-sized targets such as the legs and heads of cattle, we opted for a faster one-stage detector that is more friendly to small target detection [39].

To assess the models’ performance, we conducted experiments on the cattle body dataset using the aforementioned one-stage detector. The results demonstrated that the YOLOv5 model exhibited better performance in terms of precision, recall, *F*1 score, and mAP@0.5 compared to the other models. As a result, we opted to adopt YOLOv5 as the foundational model for our research and subsequent improvements.

The study found that incorporating attention mechanisms can effectively enhance the model’s feature extraction capability and further improve its detection and recognition capabilities for critical targets [40]. And the attention mechanism has also been proven to improve the cattle body detection model [41]. Additionally, to further validate the effectiveness of the EMA attention module, we compared it with various attention components integrated into the YOLOv5 architecture. These components include channel attention mechanisms (ECA [34], SE [35]) as well as channel and spatial attention mechanisms (CBAM [36], CA [37]). The channel attention mechanism enhances the model’s discriminative and expressive abilities for targets by weighting the features in each channel. On the other hand, the channel and spatial attention mechanism further considers the importance of each pixel position in the feature map, aiding the model in better handling images with complex structures [42]. The experimental results clearly demonstrate that YOLOv5-EMA outperforms models equipped with CBAM, SE, ECA, and CA attention units, achieving the best results. And it enhances the detection performance of the model on the legs and head of cattle. In contrast to other attention models, that either focus solely on channel information or consider both channel and spatial information without fully exploring information dependencies across diverse spatial scales, EMA attention distinguishes itself. It adeptly integrates spatial and channel attention, promoting effective information exchange among feature maps at various spatial scales. Consequently, it substantially elevates the model’s detection capabilities.

Using the same attention mechanism at different locations within a model can yield diverse outcomes [43]. Therefore, we conducted experiments to incorporate EMA attention modules at various positions in the YOLOv5 network. Through these experiments, we successfully bolstered robustness by integrating the EMA attention mechanism into the C3 module of the YOLOv5 Backbone network. This integration facilitated the aggregation of cross-spatial information across various spatial dimensions, thereby further amplifying the model’s feature representation capacity.

Above all, the YOLOv5-EMA model has showcased good performance when compared to existing models, excelling in quantitative metrics, qualitative detection outcomes, and the visualization of Grad-CAM [31] heatmaps.

While we have successfully developed a YOLOv5-EMA model for cattle body detection, there are still limitations to this study. In order to make this research practical, the model we developed must be able to adapt to actual pasture environments. However, the data we collected was captured through a mobile phone camera, whereas most pastures use fixed-angle cameras for monitoring. The purpose of using a mobile phone to collect the dataset is to capture photos from different angles, thereby enriching the composition of the cattle body dataset and improving the model’s generalization ability. But changes in shooting angles and other environmental factors may affect the model’s performance. To address this issue, we need to further optimize the model in future research to improve its robustness and enable it to process image data from actual pasture environments.

## 5. Conclusions

This paper introduces a novel model, YOLOv5-EMA, for cattle body detection, showcasing good performance in both individual cattle and local key part detection. The experimental results have demonstrated the effectiveness of YOLOv5-EMA in improving cattle body detection. The YOLOv5-EMA not only encodes information between channels to adjust the importance of them for capturing features at various scales, but also performs cross-spatial information aggregation over different spatial dimensions. This enriches feature aggregation, significantly enhancing detection performance, particularly in scenarios involving small targets and occlusions.

In our future endeavors, we will explore various data augmentation approaches to boost the robustness of the model and experiment with other attention units to capture crucial features in cattle body detection with better performance. Moreover, we will broaden our research to encompass more intricate scenarios, including the tracking and analysis of cattle behavior, in order to gain deeper insights into their patterns and characteristics. This series of work aims to promote the development of animal detection and understanding.

## Figures and Tables

**Table 1:** Data characteristics of the dataset. The label types include individual cattle, head, and legs.

Dataset	Label Type	Division Ratio	Number	Pixel Value
Training set	3	70%	5617	0~255
Test set	3	30%	2407	0~255

**Table 2:** Software and hardware configuration for experiments.

Term	Configurations
Python	3.6.9
Pytorch	1.2.0
CUDA	11.2
CUDNN	10.0.130
Hard disk	4TB SSD*3
Operating System	Ubuntu 18.04
GPU	NVIDIA GeForce GTX TITANXP
CPU	Intel Core I7 7800 X
Memory	128 GB

**Table 3:** Comparison of different models. The bolded data in the table represents the detection results using the YOLOv5-EMA model.

Model	Precision (P/%)	Recall (R/%)	*F*1 Score (*F*1/%)	Mean Average Accuracy (mAP@0.5/%)
SSD	92.3	84.2	88.0	92.2
Faster-RCNN	75.2	92.3	82.9	89.3
YOLOv2	79.0	77.0	78.0	83.1
YOLOv3	89.0	85.0	87.0	91.3
YOLOv4	88.0	87.0	87.0	92.3
YOLOv5	93.7	89.0	91.3	94.1
**Ours**	**94.8**	**90.3**	**92.5**	**95.1**

**Table 4:** Comparison of different models based on mAP (mAP@0.5/%) across different parts of cattle body. The bolded data in the table represents the detection results using the YOLOv5-EMA model.

Model	Cattle	Leg	Head
SSD	88.2	92.7	95.4
Faster-RCNN	89.3	88.1	90.5
YOLOv2	88.1	72.0	89.3
YOLOv3	92.5	89.2	92.2
YOLOv4	93.4	90.7	92.7
YOLOv5	94.8	93.4	94.0
**Ours**	**94.8**	**94.9**	**95.5**

**Table 5:** Comparison of models with or without EMA based on all criteria.The bolded data in the table represents the detection results using the EMA attention module.

Model	Precision (P/%)	Recall (R/%)	*F*1 Score (F1/%)	Mean Average Accuracy (mAP@0.5/%)
YOLOv3	89.0	85.0	87.0	91.3
**YOLOv3-EMA**	**94.5**	**88.8**	**91.8**	**93.9**
YOLOv5	93.7	89.0	91.3	94.1
**YOLOv5-EMA**	**94.8**	**90.3**	**92.5**	**95.1**

**Table 6:** Comparison of models with or without EMA based on mAP (mAP@0.5/%) across different parts of cattle body. The bolded data in the table represents the detection results using the EMA attention module.

Model	Cattle	Leg	Head
YOLOv3	92.5	89.2	92.2
**YOLOv3-EMA**	**94.2**	**93.3**	**94.3**
YOLOv5	94.8	93.4	94.0
**YOLOv5-EMA**	**94.8**	**94.9**	**95.5**

**Table 7:** Comparison of different attention models across various parts of cattle body based on mAP (mAP@0.5/%). The bolded data in the table represents the detection results using the YOLOv5-EMA model.

Model	All	Cattle	Leg	Head
YOLOv5	94.1	94.8	93.4	94.0
+CBAM	95.0	94.8	94.6	95.5
+SE	94.8	94.6	94.7	95.2
+CA	94.9	94.8	94.5	95.3
+ECA	94.9	94.8	94.5	95.5
**+EMA**	**95.1**	**94.8**	**94.9**	**95.5**

**Table 8:** Comparison of models with EMA at different position across various parts of cattle body based on mAP (mAP@0.5/%). The bolded data at position 5 in the table represents the detection results using the YOLOv5-EMA model proposed in this paper.

Model	All	Cattle	Leg	Head
Position 1	94.6	94.4	94.5	95.0
Position 2	94.5	94.4	94.1	95.0
Position 3	94.7	94.8	94.4	94.9
Position 4	94.8	94.7	94.4	95.3
**Position 5**	**95.1**	**94.8**	**94.9**	**95.5**

## Data Availability

The datasets generated and/or analyzed during the current study are available from the corresponding author on reasonable request.

## Abbreviations

The following abbreviations are used in this manuscript:

EMA	Efficient Multi-Scale Attention
YOLOv5-EMA	YOLOv5-Efficient Multi-Scale Attention
YOLOv3-EMA	YOLOv3-Efficient Multi-Scale Attention
C3EMA	C3-Efficient Multi-Scale Attention
Grad-CAM	Gradient-weighted Class Activation Mapping
RFID	Radio Frequency Identification
IOT	Internet of Things
RERP	Random Erasure and Region-visibility Prediction
YOLO	You Only Look Once
SSD	Single Shot MultiBox Detector
Faster R-CNN	Faster Region-based Convolutional Neural Networks
Mask R-CNN	Mask Region-based Convolutional Neural Networks
Conv	Convolution
CNN	Convolutional Neural Network
PCN	Part-based Convolutional Networks
1D	One-Dimensional
2D	Two-Dimensional
SPPF	Spatial Pyramid Pooling-Fast
SiLU	Sigmoid Linear Unit
BN	Batch Normalization
FPN	Feature Pyramid Network
IOU	Intersection over Union
CIOU	Complete Intersection over Union
P	Precision
R	Recall
AP	Average precision
mAP	mean Average Precision
mAP@0.5	mean Average Precision at IoU threshold of 0.5
CBAM	Convolutional Block Attention Module
SE	Squeeze-and-Excitation
CA	Coordinate Attention
ECA	Efficient Channel Attention
